# Unraveling tumor associated macrophage biology in gynecological cancers for targeted therapy in the single cell omics era

**DOI:** 10.1016/j.isci.2025.113924

**Published:** 2025-11-04

**Authors:** Shirong Zou, Shu Zhang

**Affiliations:** 1 Department of Gynecology and Obstetrics, Shanghai Key Laboratory of Gynecology Oncology, Renji Hospital, Shanghai Jiao Tong University School of Medicine, Shanghai 200127, China

**Keywords:** Immunology, Microenvironment, Oncology

## Abstract

The tumor microenvironment (TME) has long been the subject of cancer research, particularly in deciphering the complicated mechanisms underlying tumor development. Tumor associated macrophages (TAMs), as key components of the TME, exhibit remarkable heterogeneity and drive complex interactions that influence immune evasion and therapeutic resistance. Recent technological advancements, particularly single-cell RNA sequencing, have enabled the precise dissection of TAM subpopulations, offering unprecedented insights into their functional diversity. Gynecological cancers represent a global health burden with high morbidity and mortality. Despite advances in chemotherapy and immunotherapy, treatment efficacy remains suboptimal in advanced-stage patients, underscoring the urgent need to explore cellular mechanisms underlying therapeutic failure. This review aims to summarize emerging evidence on TAM subclusters in gynecological malignancies, highlight the context-dependent phenotypic plasticity of TAMs, point out the phenotype-specific roles in tumor progression and drug resistance and evaluate translational strategies targeting specific subtypes to improve clinical outcomes.

## Graphical abstract

**Figure:**
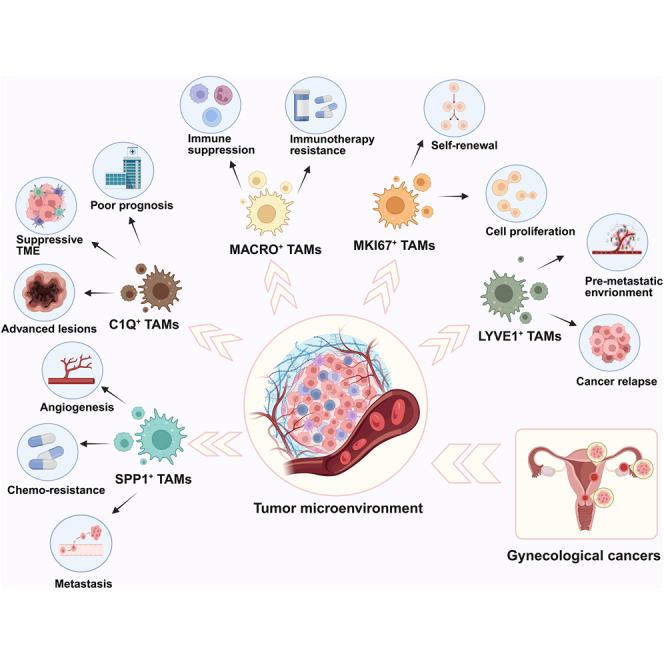

## Introduction

Macrophages are an integral component of the immune system with heterogeneity as a prominent feature—they adopt distinct phenotypes in response to genetic programs or environmental cues. Tumor-associated macrophages (TAMs), infiltrating the tumor microenvironment (TME), are also highly plastic and heterogeneous, playing a pivotal role in cancer via multiple mechanisms (e.g., promoting progression, invasion, metastasis, and treatment resistance). TAMs comprise two main compartments: tissue-resident macrophages (TRMs) and monocyte-derived infiltrating macrophages. A widely accepted classification divides TAMs into “M1” and “M2” phenotypes, based on the stimuli inducing their differentiation.

However, while the canonical M1/M2 dichotomy has provided valuable insights into TAMs in previous studies, it has proven to be insufficient when researchers have delved deeper into the dynamic and diverse nature of TAMs in contemporary investigations. Specifically, the M1 and M2 classifications merely represent two extremes of a complex functional continuum of TAMs. In real situations, their dynamic profiles encompass a spectrum ranging from transient intermediate states with numerous subgroups to highly polarized subtypes that could perform specific roles. To fully elucidate the significance of TAMs, there is a growing consensus among researchers that it is imperative to move beyond the traditional M1/M2 classification paradigm.

With the advent of single-cell multi-omics technologies, the understanding of TAMs’ sub-phenotypes has reached an unprecedented level of sophistication. Single-cell sequencing has significantly advanced the ability to dissect the intricate of TAMs, thereby offering novel insights into the composition of TAM subtypes.^1^ Additionally, spatial transcriptome analysis has facilitated the identification of TAM subtypes within defined tumor niches, further augmenting the comprehension of their spatial distribution and functional roles.^2^ Consequently, a more comprehensive understanding has been attained regarding the origins, differentiation trajectories, and site-specific functions of TAMs.

Gynecological cancers pose a significant threat to female health worldwide. For instance, the absence of effective diagnostic modalities for ovarian cancer results in presentation at advanced stages during initial clinical evaluation. More critically, resistance to chemotherapy or immunotherapy substantially impedes the clinical management of gynecological cancers. An in-depth interpretation of the diverse subtypes of TAMs and their context-dependent effects within the TME is of paramount importance for the identification of new biomarkers. These biomarkers may serve as indicators of early detection and as prognostic factors for assessing tumor malignancy. Additionally, the development of targeted therapies is being driven by novel agents designed to engage specific TAM subtypes. In this review, we seek to summarize recent advancements in the characterization of novel molecular phenotypes of TAMs in gynecological malignancies, underscoring their clinical relevance and potential applications in survival prognostication and precision medicine.

## Molecular heterogeneity of tumor associated macrophages in ovarian cancer

### Specific tumor associated macrophage subtypes and their roles in ovarian cancer progression and metastasis

Ovarian cancer (OC) is a highly complex and heterogeneous disease characterized by a diverse array of molecular and pathological subtypes. This complexity is further reflected in the TME, which is intricately composed of various cellular and molecular components. In an effort to unravel the diverse phenotypic spectra of TAMs in OC, recent research has increasingly focused on this intricate cellular subset, yielding numerous novel insights. (Table 1).

**Table 1:** Novel TAM subtypes in ovarian cancer

TAM subtypes	Gene markers	Key functional roles	Clinical implications	Reference
C1Q^+^ TAMs	*C1QA/B/C, TREM2*	1. Inhibit T cells and NK cells 2. Promote tumor progression 3. Forming suppressive TME	1. Poor prognosis 2. Advanced lesions	Brand et al.,^3^^,^^4^ Yan et al.^3^^,^^4^
CX3CR1^+^ TAMs	*CX3CR1, CCL3, CCL4, CXCL8, VEGFA, IL-1B*	1. Recruit immune cells 2. Promote angiogenesis 3. Augment Treg cells	1. Distant metastasis	Deng et al.^5^, Xu et al.^6^, Hornburg et al.^7^
CD36^+^ TAMs	*CD36, PPARG, IL-10*	1. Involved in lipid and glucose metabolism 2. Anti-inflammatory 3. Forming suppressive TME	1. Promote metastasis 2. Shorter overall survivals	Xu et al.^6^
CD11c^+^ TAMs	*CD11c, PD-L1*	1. Activate immune response 2. Induce cell death	1. Improved prognosis	Corvigno et al.,^8^^,^^9^ Kruse et al.^8^^,^^9^
LYVE1^+^ TAMs	*LYVE1, FOLR2*	1. Tumor expansion 2. Self-renewal 3. Enhance cancer cell stemness	1. Tumor relapse after omentectomy	Deng et al.,^5^^,^^10^ Zhang et al.^5^^,^^10^
TIM4^+^ TAMs	*TIM4, CD163*	1. Antigen cross-presentation 2. Immune surveillance 3. Activate CD8^+^ T cells	1. Forming pre-metastatic niche	Joshi et al.,^11^^,^^12^ Etzerodt et al.^11^^,^^12^
MMP9^+^ TAMs	*MMP9, MMP19, CXCL2, CXCL3, CTSL*	1. Represent a transitional population	1. Chemo-resistance	Deng et al.,^5^^,^^13^ Zhong et a.^5^^,^^13^
SPP1^+^ TAMs	*SPP1, CSTB, MT1G*	1. Angiogenesis 2. Tumor metabolism	1. Chemo-resistance	Jiang et al.^14^
FCN1^+^ TAMs	*MACRO, FCN1, S100A8, S100A9, CCL20*	1. Antigen presentation 2. Immune modulation 3. Pro-inflammatory	1. Response to chemotherapy	Xi et al.^15^
CCL3^+^ TAMs	*CCL3, CCL4, CCL20, CCL3L3*	1. Immune response	1. Response to chemotherapy	Xi et al.^15^
CXCL10^+^ TAMs	*CXCL10, IRF, STAT1, CSF1*	1. Recruit GZMB^+^ T cells	1. Response to immunotherapy	Ardighieri et al.^16^
CD169^+^ TAMs	*CD169, CD83, CXCL9, CXCL10, CXCL11*	1. Recruiting T cells 2. Enhance B cell population 3. Antigen presentation	1. Inhibit lymph nodes’ metastasis	Hornburg et al.^7^
MACRO^+^ TAMs	*MACRO, GPI*	1. Involved in glucose metabolism 2. A primary phenotype	1. Immunotherapy resistance	Xu et al.^6^^,^^7^ Hornburg et al.^6^^,^^7^
Siglec-9^+^ TAMs	*SIGLEC9, CD86, HLA-DR, CD163, CD206, PD-L1*	1. Immune escape 2. Related to exhausted TME	1. Worse prognosis	Wang et al.^17^
Siglec-10^+^ TAMs	*SIGLEC10*	1. Immune escape	/	Barkal et al.^18^
TREM2^+^ TAMs	*SPP1*, *C1Q*, *APOE*, *MACRO*	1. Immunosuppression 2. Related to exhausted TME	1. Advanced stages 2. Poor clinical outcomes 3. Resistance to anti-PD-1 treatment	Binnewies et al.^19^
C5aR1^+^ TAMs	*C5aR1*	1. Immunosuppression 2. Promote angiogenesis 3. Promote tumor growth	/	Binnewies et al.,^19^^,^^20^ Zhang et al.^19^^,^^20^

Representative list of TAM subtypes identified in OC. Each TAM subtype is concluded with gene markers, key functional roles, and clinical suggestions. TME: tumor microenvironment.

#### C1Q^+^ tumor associated macrophages

A distinct subset of TAMs in OC, identified as complement component 1q (C1Q)^+^ TAMs, is characterized by the high expression of *C1QA/B/C*, triggering receptor expressed on myeloid cells 2 (*TREM2*). C1Q, a key recognition molecule of the classical complement pathway, primarily exerts its functions by binding to immune complexes or other activators.^21^ C1Q^+^ TAMs represent a specific subcluster of TAMs and have been implicated in immune regulation within TME. These macrophages are believed to exert their effects through extensive interactions with other immune cells and stromal components.^3^ Specifically, C1Q^+^ TAMs have been shown to negatively regulate the activation of T cells and natural killer (NK) cells via C-type lectin domain family 2, member B (CLEC2B)- killer cell lectin like receptor B1 (KLRB1) interaction and tumor necrosis factor (TNF) signaling pathways. Additionally, C1Q^+^ TAMs communicate with fibroblasts through the expression of platelet-derived growth factor (PDGF), a process that may be linked to poor prognosis.^22^ Recent study has demonstrated that C1Q^+^ TAMs are more abundant in high-grade serous ovarian cancers (HGSCs) compared to normal tissues,^3^ suggesting their potential roles in tumor progression. Furthermore, C1Q^+^ TAMs have been shown to reprogram T cells, leading to a dysfunctional T cell infiltration profile.^23^ In other solid tumors, C1Q^+^ TAMs have been implicated in the formation of a suppressive immune microenvironment through the expression of immune checkpoint molecules,^24^ cytokines,^25^ and specific metabolites.^26^

#### CX3CR1^+^ tumor associated macrophages

Chemokine (C-X3-C motif) receptor 1 (CX3CR1)^+^ TAMs represent a distinct subcluster characterized by the high expression of various chemokines and pro-angiogenic factors, indicating their roles in immune cell recruitment and angiogenesis promotion. These TAMs are mainly enriched in inflammation-related pathways and have been shown to facilitate the progression and metastasis of OC.^5^ Single-cell transcriptomics analysis of OC tumor tissues collected from different metastatic sites revealed that CX3CR1^+^ TAMs are predominantly present in the primary tumors and peritoneal metastatic sites.^6^ Notably, CX3CR1^+^ TAMs are enriched in T cells-infiltrating tumors and can be recruited to the TME by endothelial cells and pericytes.^7^ Moreover, this subcluster can secrete Bcl2-associated athanogene 3 (BAG3), an important protein that sustains tumor growth and invasion.^27^ In colorectal cancer, CX3CR1^+^ macrophages can secrete interferon-β (IFN-β) to expand the population of regulatory T cells (Tregs), thereby contributing to tumorigenesis.^28^

#### CD36^+^ tumor associated macrophages

CD36^+^ TAMs are detectable in malignant ascites and metastatic tissues in OC, and their presence is significantly correlated with shorter overall survival.^6^ CD36, a scavenger receptor, serves as a central regulator of lipid metabolism and functions as a pro-tumoral factor in TME.^29^ CD36^+^ TAM subcluster is implicated in the maintenance of lipid and glucose homeostasis, characterized by the high expression of peroxisome proliferator-activated receptor gamma (PPARG).^6^ Additionally, CD36^+^ TAMs exhibit elevated levels of interleukin-10 (IL-10) and growth factors, indicative of their anti-inflammatory phenotype and potential role in promoting tumor progression.^6^ These TAMs are also capable of internalizing long-chain fatty acids, thereby displaying immunosuppressive properties. Given these characteristics, targeting CD36^+^ TAMs may represent an effective therapeutic strategy for inhibiting metastasis in ovarian cancer.^30^

#### CD11c^+^ tumor associated macrophages

CD11c^+^ TAMs represent a distinct subcluster of TAMs endowed with antigen-presenting capabilities, thereby playing a crucial role in innate immunity. In HGSCs, a high density of CD11c^+^ TAMs within the stromal compartment was linked to improved prognosis.^8^ This favorable outcome is likely attributed to the ability of CD11c^+^ TAMs to support immune surveillance through intimate crosstalk with CD8^+^ T cells.^8^ Furthermore, CD11c serves as a marker indicative of an activated immune profile, as it is negatively correlated with programmed death ligand 1 (PD-L1) expression.^31^ CD11c^+^ TAMs can be re-educated into a tumoricidal phenotype by CD4^+^ T cells, effectively inducing cell death at the tumor margins.^9^ However, in lung cancer, CD11c^+^ TAMs may exhibit pro-tumorigenic effects when influenced by CD4^+^ T cells via interleukin-9 (IL-9)/arginase 1 (Arg1) axis. Given these context-dependent functions, the precise roles of CD11c^+^ TAMs in OC warrant further investigation to elucidate their therapeutic potential and prognostic significance.

#### Peritoneal cavity and omentum-resident tumor associated macrophage subtypes

In OC, the absence of anatomical barriers enables the rapid dissemination of tumor cells into the peritoneal cavity, where they readily implant at metastatic sites. Besides the primary tumor, TAMs are integral components of the evolving immune microenvironment in malignant ascites and metastatic tumors. Tissue-resident macrophages (TRMs) are a specialized population of macrophages that are permanently embedded within tissues or organs. Within the peritoneal cavity, there exist two main macrophage clusters: large peritoneal macrophages (LPMs) and small peritoneal macrophages (SPMs). These macrophage populations exhibit distinct surface biomarkers and functional patterns.^32^ Generally, LPMs are more responsive to inflammatory stimuli and display a tumoricidal phenotype, whereas SPMs exhibit a more moderate response. Recent insights have further elucidated the heterogeneity of cavity-resident TAMs. Two distinct subclusters of TAMs residing in the mesothelial linings of the peritoneum have been identified. Among these, lymphatic vessel endothelial hyaluronan receptor 1 (LYVE1)^+^ TAMs, which originated from embryonic progenitors, are maintained by colony stimulating factor 1 (CSF1) secreted from stromal cells. These findings highlight the role of the TME in educating and shaping the phenotype of these macrophages. And it has been demonstrated that LYVE1^+^ TAMs represent a progressive subcluster that contributes to tumor expansion even in the absence of the omentum.^10^ Consequently, it is reasonable to speculate that LYVE1^+^ TAMs may be associated with relapse following omentectomy. In the study of Deng et al., LYVE1^+^ TAMs, characterized by the high expression of folate receptor 2 (FOLR2), were defined as TRMs and were also identified within the omentum.^5^ Among all LYVE1^+^ TAMs in the omentum, a subcluster of CD163^+^TIM4^+^ TAMs was identified. These macrophages possess self-renewal capabilities and can maintain TRM features throughout cancer progression. This subcluster enhances the stemness of cancer cells via the janus kinase (JAK)-signal transducer and activator of transcription (STAT) signaling pathway. Moreover, a study focusing on early peritoneal colonization revealed distinct functions of antigen cross-presentation in TIM4^+^ TAMs. In OC, these TAMs could engulf tumor cells after recognizing cancer antigens, thereby successfully activating CD8^+^ T cells and contributing to the accumulation of cytotoxic CD8^+^ T cells. This finding suggests a novel mechanism of immune surveillance during early metastatic condition.^11^ However, once monocyte-derived TAMs infiltrate as the predominant population, the immune surveillance defense is disrupted, rendering cancer cell depletion ineffective.^11^ In summary, these subtypes of peritoneal resident TAMs are intricately involved in forming a pre-metastatic niche conducive to tumor cell invasion and promoting the dissemination of cancer cells to distant sites.

### Tumor associated macrophages subtypes linked to the treatment of ovarian cancer

#### Tumor associated macrophages subtypes associated with chemotherapy response in ovarian cancer

TAMs play a significant role in chemotherapy resistance through various mechanisms, although the specific subpopulations contributing to this resistance remain to be fully elucidated. Recent studies have shown that the proportion of matrix metalloproteinase 9 (MMP9)^+^ TAMs increases after chemotherapy, accompanied by the upregulation of pro-inflammatory genes.^13^ These MMP9^+^ TAMs express high levels of protease genes (e.g., MMP family, cathepsin L (CTSL)) and inflammatory chemokines. Pseudo-time analysis used in single-cell genomics helps to infer the dynamic progression of TAM subtypes. Pseudo-time analysis suggests that MMP9^+^ TAMs are a transitional population that can differentiate into more mature TAM clusters, such as chemokine (C-C motif) ligand 18 (CCL18)^+^ TAMs and CX3CR1^+^ TAMs.^5^ High expression of CTSL has been identified as a risk factor in various tumors.^33^^,^^34^^,^^35^ Specifically, CTSL can promote resistance to paclitaxel and cisplatin through different mechanisms. For instance, CTSL activates the TGF-β/Smad signaling pathway to induce paclitaxel resistance, while promoting cisplatin resistance via the early growth response-1 (Egr-1)/cAMP-response element binding protein (CREB) axis.^36^ In addition to MMP9^+^ TAMs, other macrophage subpopulations, such as thrombospondin-1 (THBS1)^+^ TAMs and C-X-C motif chemokine ligand 5 (CXCL5)^+^ TAMs also experienced an increase after chemotherapy.^13^

A comparative analysis of the single-cell landscapes of patients with ovarian cancer sensitive to chemotherapy versus those resistant to chemotherapy revealed that SPP1^+^ TAMs predominate in chemo-resistant patients. The top expressed genes in SPP1^+^ TAMs include resistance-related genes and those involved in angiogenesis and tumor metabolism. These macrophages interact with tumor cells via SPP1/CD44 axis.^14^ Upon receiving the SPP1 signal from TAMs via the CD44 receptor, tumor cells activate the integrin and phosphodiesterase 3B (PDE3B) pathways, ultimately inducing chemotherapy resistance.^37^ In contrast, patients responsive to chemotherapy exhibit a higher proportion of ficolin 1 (FCN1)^+^ TAMs and chemokine (C-C motif) ligand 3 (CCL3)^+^ TAMs.^15^ FCN1^+^ TAMs co-express *MACRO*, S100A8 and *CCL20* and display a pro-inflammatory phenotype. Functional analysis indicates that these TAMs are associated with phagocytosis, antigen presentation, and immune modulation. CCL3^+^ TAMs, on the other hand, highly express chemokine genes and are involved in immune response pathways.

#### Tumor associated macrophage subtypes involved in immunotherapy of ovarian cancer

Over the past years, immunotherapy has shown promising efficacy in patients with OC, with notable advancements in immune checkpoint blockade (ICB), adoptive cell therapy (ACT) and chimeric antigen receptor T (CAR-T) cell therapy. However, the success of immunotherapy largely depends on the tumor immune profile, as so-called “cold” tumors with an immunosuppressive TME often exhibit resistance to these therapies. To improve the therapeutic outcomes of immunotherapy, a deeper understanding of the immune status of the TME, particularly the role of TAMs, is essential. This knowledge can help identify potential biomarkers and therapeutic targets to overcome immunosuppression and enhance the efficacy of ICB in patients with OC.

Specific TAM subclusters are related to the T cell infiltration landscapes in OC. For example, C-X-C motif chemokine ligand 10 (CXCL10)^+^ TAMs exhibit a positive correlation with T cell infiltration in HGSCs.^16^ High infiltration of CXCL10^+^ TAMs is correlated to improved clinical outcomes and enhanced responsiveness to immunotherapy. The CXCL10^+^ TAM subcluster is induced by IFN-γ, which is secreted by T cells and NK cells. In turn, CXCL10^+^ TAMs recruit Granzyme B (GZMB)^+^ T cells into the TME, establishing a positive immune response circuit.^16^ Conversely, ovarian clear cell carcinomas (CCCs) exhibit limited CXCL10^+^ TAM infiltration, resulting in poor T cell infiltration and a moderate response to immunotherapy.^16^ CXCL10 derived from TAMs has been demonstrated to be essential for ICB therapy; the depletion of CXCL10 significantly diminishes the efficacy of ICB.^38^ In another study, a mature TAM subcluster characterized by CD169 expression was found to recruit T cells via the CXCL9/10/11-CXCR3 axis. CD169^+^ TAM cluster are predominantly observed in “excluded” and “infiltrated” tumors.^7^ These macrophages play protective roles in metastatic lymph nodes by enhancing B cell populations and initiating anti-tumor immune responses.^39^ Additionally, CD169^+^ TAMs function as antigen-presenting cells. During tumor invasion into draining lymph nodes, the suppression of CD169^+^ TAM cluster is the initial step in establishing a pre-metastatic environment.^40^

On the contrary, in the “immune desert” TME characterized by sparse T cell infiltration, the predominant TAM subcluster is MACRO^+^ TAMs. These macrophages are not only present *in situ* within the tumor but are also detected in lymph nodes and ascites cells. This subcluster is considered a primary phenotype during the differentiation process from monocytes to macrophages. MACRO^+^ TAMs express glucose-6-isomerase (GPI), indicating the activation of the glycolysis pathway in this cluster.

Other subclusters have also been identified as key contributors to the immunosuppressive TME. For instance, sialic acid-binding Ig-like lectin 9 (Siglec-9)^+^ TAMs engage in intense crosstalk with CD8^+^ T cells, either directly or indirectly, thereby promoting tumor immune escape and contributing to an exhausted TME. Siglec-9^+^ TAM subcluster represents a terminally differentiated state with pro-inflammatory programs and secretes immunosuppressive factors such as PD-L1 and IL-10. High infiltration of this subtype can impair the immune response of CD8^+^ T cells, correlating with a worse prognosis.^17^ The strong correlation between Siglec-9 expression and immune infiltration status has been confirmed in many other tumor types.^41^ Therapeutically, Siglec-9^+^ TAMs represent a potential biomarker for predicting responsiveness to ICB therapy. Moreover, recent studies have validated that the combination of PD-1 and Siglec-9 blockade can enhance therapeutic efficiency.^17^ Moreover, sialic acid-binding Ig-like lectin 10 (Siglec-10)^+^ TAMs could promote immune escape through interactions with CD24 signals derived from tumor cells.^18^ Studies have confirmed that the depletion of either CD24 or Siglec-10 can reinvigorate the phagocytosis of TAMs and reduce tumor growth. In OC, TREM2^+^ TAMs are enriched in the exhausted TME and are associated with disease stages and poor clinical outcomes.^19^ TREM2^+^ TAMs are linked to an exhausted immune state and resistance to anti-PD-1 treatment. TREM2 is a transmembrane receptor and is considered a marker of TAMs with the high expression of *SPP1*, *C1Q*, *APOE*, and *MACRO*.^42^ Of note, TREM2^+^ TAMs exhibit a lipid-associated macrophage (LAMs) signature^43^ and accumulate lipid droplets, which may underlie their immunosuppressive role.

In HGSCs, complement component 5a receptor 1 (C5aR1)^+^ TAMs have been explored as potential negative regulators of immune responses and promoters of angiogenesis.^20^ C5aR1, the receptor of complement C5a, plays a pivotal role in tumor progression by promoting an immunosuppressive TME through the C5a/C5aR1 axis.^44^ This axis activates pro-tumoral signaling pathways and cytokine production, thereby fostering tumor growth and resistance to therapy. C5aR1^+^ TAMs contribute to the formation of a suppressive microenvironment by interacting with CD8^+^ T cells and Tregs. These interactions enhance Treg function and IL-10 expression while impairing the cytotoxic activity of CD8^+^ T cells.^20^ This dual mechanism effectively suppresses antitumor immunity and promotes tumor progression.

In addition, the SPP1^+^ TAMs mentioned above not only contribute to chemo-resistance but also negatively regulate the responsiveness to immunotherapy. A study found that SPP1^+^ TAMs drive T cell exhaustion through the SPP1/CD44 axis, forming a suppressive TME.^45^

Currently, more feasible therapeutic strategies targeting TAMs in OC are under investigation. One promising approach involves the blockade of common lymphatic endothelial and vascular endothelial receptor-1 (Clever-1)^+^ TAMs, a maneuver that can initiate adaptive immunity and upregulate the expression of IFN signals and CXCL10.^46^ Furthermore, eliminating Clever-1 has been demonstrated to enhance antigen presentation and augment the secretion of pro-inflammatory cytokines such as TNF-α, interleukin-12 (IL-12).^47^ This strategy is predicted on the observation that anti-Clever-1 antibodies can activate lymphocytes while downregulating negative immune checkpoint molecules.^48^ Notably, targeting this subcluster might yield particular efficacy in non-inflamed “cold” tumors that lack pre-existing IFN infiltration.^46^ Moreover, FP-1305 (bexmarilimab), an anti-Clever-1 antibody, has shown favorable therapeutic efficacy in patients with advanced solid tumor.^48^^,^^49^

Given the essential roles of SPP1^+^ TAMs in chemotherapy and immunotherapy, therapeutic inhibitors of SPP1 have been developed. A nano-formulation (CANDI460) that specifically targets SPP1^+^ TAMs has shown synergistic effects both *in vitro* and *in vivo*.^50^ Additionally, targeting TREM2^+^ TAMs to improve the immunosuppressive TME represents an emerging trend. Researchers have demonstrated enhanced therapeutic efficacy by combining anti-TREM2 and anti-PD-1 therapy in OC models. The humanized anti-TREM2 monoclonal antibody PY314 is currently being tested in clinical cohorts.^19^ The combination effects of PY314 and pembrolizumab have also been evaluated in subjects with platinum-resistant. However, the results suggested that it warrants further confirmation in patients with heavily platinum resistance.^51^

## Molecular subtypes of tumor associated macrophages in cervical cancer

### Distinct tumor associated macrophage subpopulations in cervical cancer pathogenesis

#### HPV^+^ tumor associated macrophages

It is widely accepted that human papillomavirus (HPV) infection is an etiological factor for cervical cancer (CC), and emerging evidence shows HPV also modulates TAM phenotypes. In a study by Wang et al., single-cell transcriptomic analysis was employed to confirm the expression of HPV genes on TAMs.^52^ Specifically, HPV16^+^ TAMs express high levels of anti-tumor genes such as *IQCB1* and *PDZD11*. Functionally, the HPV16^+^ TAM cluster was enriched in cellular adhesion pathways, indicating an enhanced characteristic to invade through tissue barriers. Moreover, HPV16^+^ TAMs may possess the ability to recruit more immune cells into the TME. Intriguingly, HPV16^+^ TAMs were associated with favorable prognosis, highlighting their complex relationship with tumor progression.^52^

#### Metastasis-promoting tumor associated macrophages

SPP1^+^ TAMs represent a key subset linked to metastatic progression. Co-express *MACRO*, *TMSB4X*, *TMSB10,* and *CSTB*, these macrophages enrich pathways related to extracellular matrix (ECM)-receptor signaling and tumor metabolism. Notably, studies have demonstrated that SPP1^+^ TAMs exhibit high infiltration in metastatic lymph nodes.^53^ Extensive crosstalk with other cellular components within the TME is a hallmark of this cluster. Specifically, SPP1^+^ TAMs can adhere to stromal cells and participate in angiogenesis and EMT pathways,^54^ which may underlie their role in promoting metastasis. Additionally, SPP1^+^ TAMs interact with cancer cells via the SPP1-CD44 signaling axis, an interaction that is more pronounced in high-grade squamous intraepithelial lesion (HSIL) and CC samples compared to low-grade squamous intraepithelial lesion (LSIL) samples.^55^ Furthermore, SPP1^+^ TAMs can engage with Tregs and attract them into the TME through specific chemokine pairs, promoting immunosuppression.^54^

Concurrently, C1Q^+^ TAM subcluster has been identified in CC and plays a critical role in disease progression. Within this subcluster, C1Q^+^ TAMs in CC can be further subclassified into C1QA^+^ TAMs and C1QC^+^ TAMs. C1QA^+^ TAMs presented high levels of *MACRO*, *APOE*, *CTSD*, *CXCL10*, and also antigen-presenting genes.^53^ This subcluster is involved in various immune modulation processes and cytokine signaling pathways. Given their presence in both primary tumors and metastatic lymph nodes, further exploration has revealed differences between C1QA^+^ TAMs at these different sites. The predominant phenotype in the primary tumor is C1QA^+^MRC1^low^ macrophages, whereas the enriched phenotype in metastasis lymph nodes is C1QA^+^MRC1^high^ macrophages.^56^ C1QA^+^MRC1^high^ TAMs possess an immunosuppressive subtype with functions related to phagocytosis and immune activation. This discrepancy highlights their potential role in CC metastasis. Moreover, C1QC^+^ TAMs in CC could highly express *TREM2* and MHC class Ⅱ genes and are mainly enriched in pathways related to complement activation, antigen presentation, and phagocytosis.^57^ As C1Q is a vital factor in initiating the complement cascade, C1Q^+^ TAMs can trigger the complement cascade response, thereby accelerating tumor growth.

S100 calcium binding protein A8 (*S100A8*)^+^ TAMs co-express *S100A8/2/6*, *CSTA*, and *CSTD* and were predominantly localized in metastatic lymph nodes.^53^ Notably, there are plasma cell markers expressed on S100A8^+^ TAMs, suggesting a potential interaction between TAMs and plasma cells in shaping the pre-metastatic environment. S100A8 is a member of the S100 protein family, which is known for its rapid response to inflammatory stimuli.^58^ Accumulating evidence suggested that S100A8^+^ TAMs could promote cancer cell proliferation and evasion through the nuclear factor kappa B (NF-κB) signaling pathway.^59^

Compared to the traditional M1/M2 TAM signatures, two distinct TAM subclusters (SPP1^+^ TAMs and C1QC^+^ TAMs) have been identified as more clinically informative in CC.^54^ The SPP1/C1QC signatures could effectively predict tumor stages and clinical outcomes. Patients with higher *C1QC* gene signatures tend to present with the lower International Federation of Gynecology and Obstetrics (FIGO) stages and longer overall survival, while the opposite is observed in patients with higher *SPP1* gene signatures.^60^ Furthermore, the SPP1/C1QC signatures are closely correlated with TME infiltration and the immune landscape. Studies have shown that *C1QC*^high^ groups can recruit more immune cells, forming an “immune-hot” TME, whereas *SPP1*^high^ groups are associated with an “immune-cold” TME.^60^ This discrepancy can be leveraged to inform the responsiveness to immunotherapy. Additionally, *C1QC*^high^ groups exhibit higher expression of immune checkpoints compared to *SPP1*^high^ TAMs.

#### Pro-tumoral tumor associated macrophages

THBS1^+^ TAMs co-express genes such as *VCAN*, *TIMP1*, *IL1B*, *FCN1*, and cellular adhesion-related genes, including S100A8 and S100A9. These THBS1^+^ TAMs found in CC presented an anti-inflammatory phenotype.^53^ Trajectory analysis suggests that this subcluster represents a differentiation endpoint distinct from C1QA^+^ TAMs. Targeting THBS1 reverses immune suppression mediated by TAMs, and reactivates cytotoxic T cells.^61^ High expression of THBS1 is associated with adverse prognosis in CC.^53^

Marker of proliferation Ki-67 (MKI67)^+^ TAMs have been identified as a unique subcluster with the high expression of cell proliferation genes, including *MKI67*, *TPO2A*, *TYMS,* and *STMN1*. These macrophages are considered to possess self-renewal ability,^57^ as evidenced by their high expression of the mini chromosome maintenance (MCM) protein family genes, which are specifically associated with cycling cells.^53^ MKI67^+^ cancer cells^62^ and MKI67^+^ T cells^58^ have both been shown to exhibit strong proliferative ability, contributing to cancer development. However, the precise roles of MKI67^+^ TAMs in CC progression remain to be fully confirmed.

#### Lipid-associated macrophages

Apolipoprotein E (APOE)^+^ TAMs frequently exhibit high expression levels of *APOE*, *APOC1*, *SPP1,* and *GPNMB*. Similar to those identified in other solid tumors, APOE^+^ TAMs that existed in CC can be characterized as lipid-associated macrophages (LAMs). These LAMs participate in various immune response processes and lipid metabolism, particularly through the TREM2 receptor,^63^ which is crucial for their functions. LAMs can be recruited by cancer-associated fibroblasts (CAFs) and subsequently restrict T cell functions, thereby contributing to the immunosuppressive TME.^64^ LAMs represent a pro-inflammatory subtype with high angiogenesis scores. Additionally, alterations in N6-methyladenosine (m6A) modifications within LAMs may lead to changes in TME.^65^ In conclusion, LAMs function as a pro-tumoral factor and may serve as a potential biomarker for predicting the malignant progression of CC.

#### Others

C-C motif chemokine ligand 20 (CCL20)^+^ TAMs were identified in advanced CC samples and were negatively associated with clinical outcomes.^65^ Fc fragment of IgG receptor IIIa (FCGR3A)^+^ TAMs and CD68^+^ TAMs were also identified in cervical adenocarcinoma samples.^66^ FCGR3A^+^ TAMs highly expressed chemokine genes such as *CCL18*, *CCL13*, *CXCL5*, and *CCL18*. A subcluster of FCGR3A^+^ TAMs identified in gastric cancer was relevant to worse prognosis and reduced response to immunotherapy.^67^ High expression of FCGR3A was also associated with immune checkpoint molecules and poor overall survival in glioma.^68^ CD68, a pro-tumoral TAMs marker, and linked to suppressive functions of TAMs. High infiltration of CD68^+^ TAMs was significantly correlated with T cell infiltration, tumor invasion, and other malignant characteristics.^69^

### Therapeutic implications of tumor associated macrophage subtypes in cervical cancer

TAM subtypes exhibit distinct distributions across CC pathological types. Squamous cell carcinoma (SCC) is the most common type. Compared to adenocarcinoma (AD), the predominant cluster in SCC was CD74^+^ TAMs.^66^ This cluster expresses MHC class Ⅰ molecules and cytokines such as *CXCL9* and *CXCL10*. CD74^+^ TAMs exclusively express CD74, a transmembrane glycoprotein involved in antigen presentation and acting as the receptor for macrophage migration inhibitory factors (MIFs). High expression of human leukocyte antigen (HLA) molecules in these TAMs can activate cytotoxic T cells via co-stimulatory molecules. Researchers have found that CD74^+^ TAMs are upregulated after platinum-based neoadjuvant chemotherapy (NACT), which may lead to weakened efficacy of NACT. In CC, CD74^+^ TAMs have intense crosstalk with malignant cells, contributing to pro-inflammatory phenotypes and abrogated phagocytic ability. Blockade of CD74^+^ TAMs could be a promising strategy to enhance the effectiveness of NACT treatment.

The heterogeneity of TAMs in CC underscores their multifaceted roles in tumor initiation, progression, and metastasis (Table 2). Pathological-type-specific distributions, such as CD74^+^ TAMs in SCC, highlight the need for subtype-driven therapeutic approaches. Future research should prioritize mechanistic insights into TAM crosstalk and validate subtype-specific interventions in clinical trials.

**Table 2:** Novel TAM subtypes in cervical cancer

TAM subtypes	Gene markers	Key functional roles	Clinical implications	Reference
HPV^+^ TAMs	*E1*, *E6*, *E7*, *IQCB1*, *PDZD11*, *WAS*, *MYO1F*	1. Anti-tumor roles 2. Cellular adhesion 3. Recruiting immune cells	1. Better prognosis	Wang et al.^52^
SPP1^+^ TAMs	*SPP1*, *MACRO*, *TMSB4X*, *TMSB10*, *CSTB*	1. Angiogenesis 2. EMT process 3. Recruit Treg cells	1. Lymph nodes metastasis	Li et al.,^53^^,^^54^^,^^56^^,^^60^ Qiu et al.,^53^^,^^54^^,^^56^^,^^60^ Li et al.,^53^^,^^54^^,^^56^^,^^60^ Liet al.^53^^,^^54^^,^^56^^,^^60^
C1Q^+^ TAMs	*C1Q, MACRO, APOE, CTSD, CXCL10, TREM2*	1. Phagocytosis 2. Immune modulation 3. Initiate the complement cascade	1. Tumor metastasis	Li and Hua,^53^^,^^56^^,^^57^^,^^60^ Li et al.,^53^^,^^56^^,^^57^^,^^60^ Cao et al.,^53^^,^^56^^,^^57^^,^^60^ Li et al.^53^^,^^56^^,^^57^^,^^60^
THBS1^+^ TAMs	*VCAN, TIMP1, IL1B, FCN1*	1. Anti-inflammatory 2. Immune suppression 3. Inhibit cytotoxicity of T cells	1. Poor prognosis	Li and Hua^53^
MKI67^+^ TAMs	*MKI67, TPO2A, TYMS, STMN1, MCM4, MCM5, MCM7*	1. Cell recycling 2. Self-renewal	/	Li and Hua^53^
S100A8^+^ TAMs	*S100A8/2/6, CSTA, CSTD*	1. Promote cancer proliferation and invasion	1. Cancer metastasis	Li and Hua,^53^^,^^56^ Li et al.^53^^,^^56^
APOE^+^ TAMs	*APOE, APOC1, SPP1, GPNMB*	1. Lipid metabolism 2. Pro-inflammatory 3. Immune response	/	Liu et al.^65^
CD74^+^ TAMs	*CD74, CXCL9, CXCL10*	1. Antigen presentation 2. T cells activation 3. Pro-inflammatory	1. Impairing the efficacy of NACT	Li et al.,^66^ Wang et al.^66^^,^^70^

Representative list of TAM subtypes identified in CC. Each TAM subtypes is concluded with gene markers, key functional roles, and clinical suggestions. HPV: human papillomavirus; TME: tumor microenvironment. EMT: epithelial-mesenchymal transition. NACT: neoadjuvant chemotherapy.

## Molecular heterogeneity of tumor associated macrophages in endometrial cancer

### SPP1^+^ tumor associated macrophages

SPP1^+^ TAMs exhibited a higher proportion in endometrial cancer (EC) samples compared to normal tissues.^71^ In EC, SPP1^+^ TAMs were characterized by the high expression of chemokines such as *CXCL8*, *IL1B*, *CCL3*, *THBS1*. SPP1^+^ TAMs in EC are highly enriched in lymphocyte activation pathways, indicating their role in facilitating other immune cellular components. In terms of cellular crosstalk, SPP1^+^ TAMs express *SPP1* and nicotinamide phosphoribosyl transferase (*NAMPT*) to interact with the stroma and cancer cells. And the main receptor on SPP1^+^ TAMs is CD74.^71^ Furthermore, the study also showed that SPP1^+^ TAMs were enriched in EC with phosphatase and tensin homolog (PTEN) and TP53 mutations, suggesting their involvement in the tumorigenesis mechanisms of PTEN mutation-related EC.^71^

### C1Q^+^ tumor associated macrophages

C1QC^+^ TAMs identified in EC are highly expressed *C1Q* with the low expression of *IL1B*. However, this same cluster could exhibit different functional patterns in tumor and normal contexts.^71^ In EC samples, C1Q^+^ TAMs were associated with various malignant features, while in normal tissues, C1Q^+^ TAMs participated primarily in metabolic processes. In addition, C1Q^+^ TAMs in normal tissue presented fewer crosstalk with other cells.^71^ Consequently, these findings suggested that TME could shape TAM clusters and educate them to pro-tumoral phenotypes. Pseudo-time analysis suggested that C1QC^+^ TAMs may derive from oxidized low-density lipoprotein receptor 1 (OLR1)^+^ TAMs and can also differentiate into MACRO^+^ TAMs.^72^

### LYVE1^+^ tumor associated macrophages

In EC, the LYVE1^+^ TAM cluster is featured by the high expression of complement C3 (*C3*) and low expression of *IL-1B*. LYVE1^+^ TAMs identified in EC have high expression of several autoimmunity-related genes and are enriched in complement and HLA-related pathways.^71^ As a result, this cluster likely plays a key role in antigen presentation and maintenance of the immune environment within the TME. LYVE1^+^ TAMs are considered a subcluster of tissue-resident TAMs, which could be identified in both EC samples and normal endometrial tissues.

### Cycling tumor associated macrophages

Cycling TAMs have also been identified in EC and are featured by the high expression of several key genes, including *MKI67*, *TYMS*, *TOP2A,* and *CENPF*.^73^^,^^74^ *MKI67* encodes the Ki-67 protein, a well-established marker of cell proliferation, which has been widely used as a prognostic biomarker in various malignancies.^75^^,^^76^ Thymidylate synthase (*TYMS*), identified as an oncogene, is known to promote tumorigenesis. Its inhibition has been shown to not only suppress the progression of cancer cells but also enhance sensitivity to chemotherapy.^77^ Topoisomerase (DNA) II alpha (*TOP2A*) is a DNA topoisomerase enzyme, playing a crucial role in maintaining the integrity of DNA fragments. And its overexpression during cell division has been implicated in carcinogenesis.^78^ Additionally, centromere protein F (*CENPF*) has been shown to promote tumor metastasis and angiogenesis through the FAK/MAPK signaling pathway.^79^ Despite these findings, the specific roles of cycling TAMs in EC remain to be fully elucidated.

### APOC^+^ tumor associated macrophages

Apolipoprotein C (APOC)^+^ TAMs express high levels of *APOC1*, *AOPE,* and *IFITM3*, and are closely related to interferon pathways, endocytosis, and immune regulation.^74^ These APOC^+^ TAMs have been implicated in various immunological processes, highlighting their multifaceted roles within the TME. APOC^+^ TAMs have been reported to correlate with immune infiltration and can promote cancer metastasis through the secretion of C-C motif chemokine ligand 5 (CCL5).^80^ Conversely, within the APOC family, APOC3 has been shown to enhance the anti-tumor activity of CD8^+^ T cells by activating TAMs, which is related to improved survival outcomes.^81^ These findings indicate that APOC^+^ TAMs may exhibit both anti-tumoral and pro-tumoral functions, depending on the specific context and signaling pathways involved.

### MACRO^+^ tumor associated macrophages

In EC, MACRO^+^ TAMs express high levels of CD68 and APOE.^72^ Studies have confirmed that the capacity of MACRO^+^ TAMs to produce TNF-α is impaired in response to LPS/INF-γ stimulation, suggesting a relatively moderate responsiveness to inflammatory stimuli.^82^ And MACRO^+^ TAM subcluster is considered an immunosuppressive subtype.

### Others

FCN1^+^ TAMs found in EC are mainly enriched in pathways related to cytokine production and positive immune responses.^73^ Specifically, FCN1^+^ TAMs are capable of differentiating into C1Q^+^ TAMs and SPP1^+^ TAMs, thereby contributing to the phenotypic diversity of the TAM population.^25^ Regarding OLR1^+^ TAMs, OLR1 functions as a scavenger receptor with oxidized low-density lipoprotein (ox-LDL) as a critical ligand. It has been implicated in both inflammatory diseases and cancers.^83^ CXCL3^+^ TAMs are characterized by high expression levels of chemokines, including CXCL2, CXCL1, CXCL3.^74^ Studies have demonstrated that this subcluster is exclusively present in tumor samples and is primarily correlated to cytokine responses and the MAPK cascade.^74^ GZMA^+^ TAMs represent a cluster related to leukocyte activation, with the high expression of cytotoxicity-associated genes such as *NKG7*, *GZMA*, and *GZMB*. Survival analysis has suggested that the presence of GZMA^+^ TAMs is associated with a better prognosis.^74^ Moreover, S100A8^+^ TAMs in EC highly express FCN1 and S100A12 and are primarily enriched in the vascular endothelial growth factor A (VEGFA)-vascular endothelial growth factor receptor 2 (VEGFR2) signaling pathway and cytokine production responses.^74^ Collagen alpha-1 (I) chain (COL1A1)^+^ TAMs exhibit high expression of collagen family members, such as *COL1A1*, *COL1A2*, and *COL3A1*. Pseudo-time analysis has revealed the dynamic transitions among these TAM subclusters. APOC1^+^ macrophages represent an early-stage cluster, while GZMA^+^ TAMs, MK167^+^ TAMs, and COL1A1^+^ TAMs are present at the intermediate stage. And these subclusters ultimately differentiate into CXCL3^+^ TAMs and S100A8^+^ TAMs.

Therapeutically, a study has demonstrated that the high infiltration of SPP1^+^ TAMs is related to chemotherapy resistance, while it may positively correlate with responsiveness to immunotherapy.^71^ Moreover, the C1Q^+^ TAM cluster has been found to be associated with resistance to docetaxel.^71^ Regarding immunotherapy, targeting the MACRO^+^ TAM subcluster with antibodies has exhibited substantial anti-tumoral efficacy and the potential to enhance the response of checkpoint therapy. While new subtypes of TAMs have been identified in EC (Table 3), corresponding research remains limited. And the precise functional roles of these subtypes require further validation through robust *in vitro* and *in vivo* experiments, building upon insights derived from single-cell sequencing. Such investigations would provide a solid foundation for advancing the development of subtype-specific targeting agents.

**Table 3:** Novel TAM subtypes in endometrial cancer

TAMs subtype	Gene markers	Key functional roles	Clinical implications	Reference
SPP1^+^ TAMs	*CXCL8, IL1B, CCL3, THBS1*	1. Angiogenesis 2. Tumor growth 3. Tumor metastasis 4. Lymphocytes activation	1. Chemotherapy resistance	Wu et al.,^71^^,^^73^ Ren et al.^71^^,^^73^
C1Q^+^ TAMs	*C1Q*	1. Pro-tumoral effects	1. Resistance to docetaxel	Wu et al.,^71^^,^^72^ Ren et al.^71^^,^^72^
LYVE1^+^ TAMs	*LYVE1, C3*	1. Antigen presentation 2. Activating complement pathways	/	Wu et al.^71^
Cycling TAMs	*MKI67, TYMS, TOP2A, CENPF*	1. Cell proliferation	/	Ren et al.,^73^^,^^74^ Ren et al.,^73^^,^^74^
APOC^+^ TAMs	*APOC1, APOE, IFITM3*	1. Immune infiltration 2. Tumor metastasis	/	Ren et al.^74^
MACRO^+^ TAMs	*CD68, APOE*	1. immunosuppression	/	Guo et al.^72^

Representative list of TAM subtypes identified in EC. Each TAM subtypes is concluded with gene markers, key functional roles, and clinical suggestions.

## Conclusion and prospects

**Figure 1:**
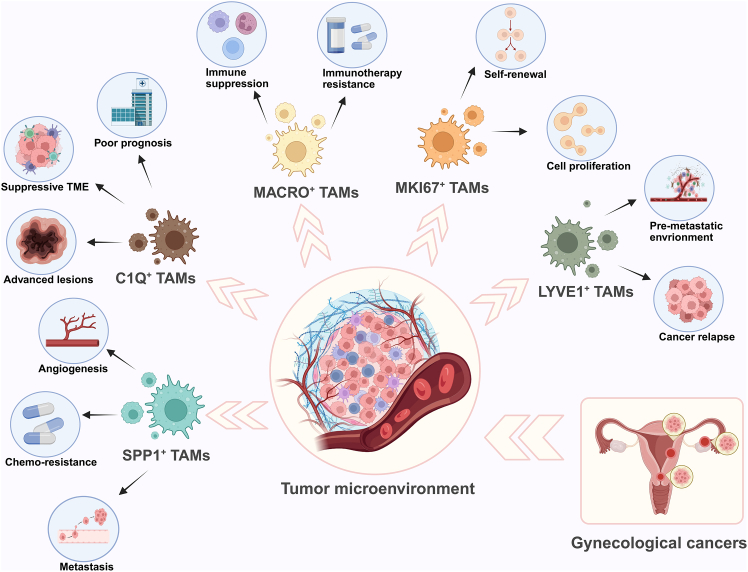
Some common TAM subtypes presented in gynecological cancers and their potential roles This schematic diagram illustrates the diverse roles of tumor-associated macrophages (TAMs) subtypes in the tumor microenvironment (TME) of gynecological cancers. SPP1^+^ TAMs are implicated in angiogenesis, metastasis, and chemo-resistance. C1Q^+^ TAMs are linked to the development of advanced lesions, formation of a suppressive TME, and poor prognosis. MACRO^+^ TAMs are involved in immune suppression and contributing to immunotherapy resistance. MKI67^+^ TAMs are mainly associated with self-renewal and cell proliferation processes. LYVE1^+^ TAMs play a role in forming the pre-metastatic environment and are related to cancer relapse. (Created in https://BioRender.com.).

The conception of macrophage states has traversed through the era of oversimplification and the era of deconstruction. The comprehension of TAMs has transcended the classical M1/M2 dichotomy, reaching unprecedented depth based on granular classification. Current research on TAMs in gynecological cancers has uncovered a diverse array of novel subtypes (Figure 1). Notably, many of these newly identified TAM subtypes have also emerged in studies of other solid tumors, suggesting conserved biological functions across different cancer types. Specifically, SPP1^+^ TAMs have emerged as key regulators of angiogenesis and metastasis, which positions this subtype as a promising biomarker for predicting distant metastasis. Targeting this subtype by specific antibody may overcome chemotherapy resistance. Due to the relationship between C1Q^+^ TAMs and suppressive TME, patients with high levels of C1Q^+^ TAMs present with advanced-stage lesions and exhibit poor prognosis. These findings underscore the critical roles of distinct TAM subtypes in gynecological cancer progression and highlight their potential as therapeutic targets.

**Figure 2:**
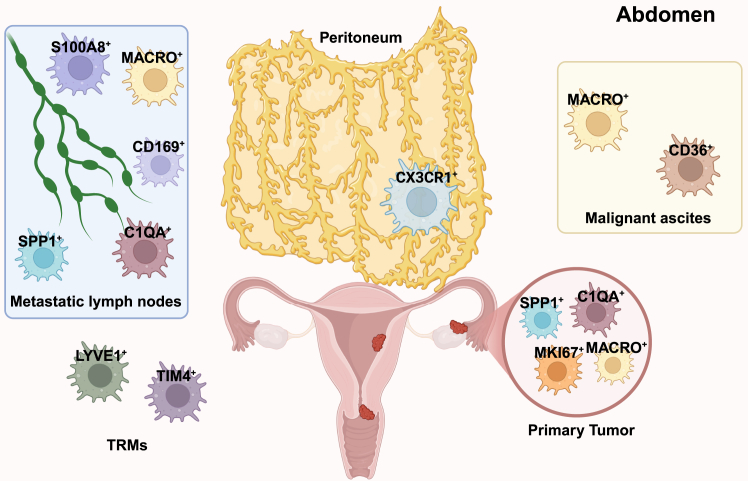
Spatial distributions of TAM subtypes in gynecological cancer Most TAM subtypes are predominantly localized in primary tumors, including SPP1^+^ TAMs, C1Q^+^ TAMs, MKI67^+^ TAMs and MACRO^+^ TAMs. Concurrently, the presence of SPP1^+^ TAMs, S100A8^+^ TAMs, MACRO^+^ TAMs, CD169^+^ TAMs and C1QA^+^ TAMs in metastatic lymph nodes suggests their potential involvement in tumor metastasis. In the peritoneum, CX3CR1^+^ TAMs are identified. As tissue-resident macrophages (TRMs), LYVE1^+^ TAMs and TIM4^+^ TAMs represent the two major populations in the abdominal cavity. Additionally, MACRO^+^ TAMs and CD36^+^ TAMs are detectable in malignant ascites. (Created in https://BioRender.com.).

**Figure 3:**
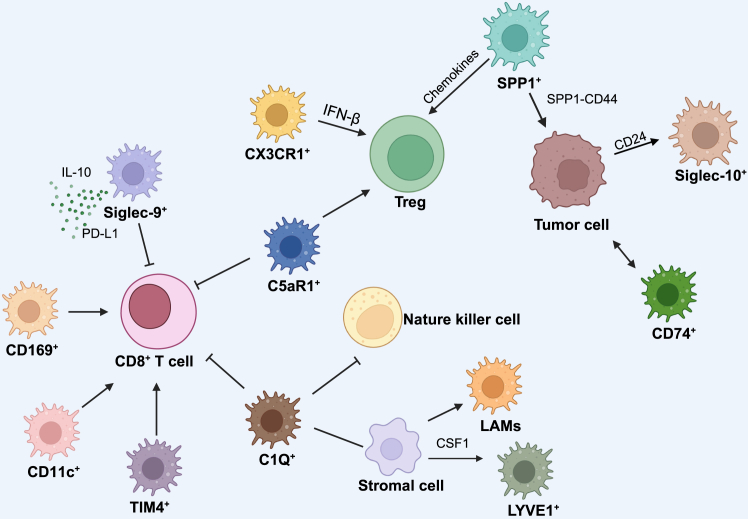
Crosstalk of TAM subtypes and other cellular components This diagram illustrates the complex cellular interactions within the tumor immune microenvironment, focusing on the crosstalk between TAMs and various immune cells or tumor cells in the context of gynecological cancers. CD8^+^ T cells are inhibited by Siglec9^+^ TAMs and CX3CR1^+^ TAMs, C5aR1^+^ TAMs and C1Q^+^ TAMs. CD8^+^ T cells are positively regulated by CD169^+^ TAMs, CD11c^+^ TAMs and TIM4^+^ TAMs. Regulatory T cells (Treg) are recruited by chemokines from SPP1^+^ TAMs, contributing to immune regulation. Tregs are activated by CX3CR1^+^ TAMs, C5aR1^+^ TAMs. Stromal cells secrete CSF1 to influence LYVE1^+^ TAMs and activate lipid-associated macrophages (LAMs). SPP1^+^ TAMs, CD74^+^ TAMs and Siglec10^+^ TAMs communicate with tumor cells in different pathways. (Created in https://BioRender.com.).

A key point in tumor biology is that TAM phenotypes have context-driven plasticity. For instance, CD11c^+^ TAMs exhibit anti-tumor and pro-tumor states, governed by contextual cues from the different TME. Moreover, even within a single TAM subtype, divergent functional roles may be observed across normal tissues, primary tumors, and metastatic lymph nodes. The high resolution of scRNA-seq has revealed that distinct TAM subtypes, dominant in spatially segregated niches, exert spatially specific functions (Figure 2). TAM subtypes disseminated in metastatic lymph nodes, peritoneal cavities, and malignant ascites contribute to the establishment of a pre-metastatic niche, thereby facilitating tumor invasion. Additionally, TAM subtypes undergo dynamic shifts in population composition after chemotherapy or immunotherapy, a phenomenon that may partially explain treatment resistance. Moreover, the intense communication of TAMs with other cancer cells or immune cells indicates the complicated roles they could play (Figure 3).

Despite significant advancements in the study of TAMs, several limitations persist within this research domain. One of the most notable challenges is the lack of a standardized annotation method for TAMs. This inconsistency results in the same TAM cluster being defined as distinct clusters across different studies, leading to potential misinterpretations and difficulties in integrating findings. Moreover, while lots of research has provided valuable insights into the complex spectrum of TAMs, these resources often fail to fully capture the spatial and temporal heterogeneity of these cells within the TME. This gap highlights the need for more comprehensive and standardized approaches to describe and analyze TAMs across various cancer types and stages.

The favorable outcomes of immunotherapy in many kinds of solid tumors have reinvigorated interest in targeting TAMs as a novel therapeutic strategy. Current strategies for targeting TAMs encompass eliminating TAMs, inhibiting their recruitment to tumor sites, and reprogramming their phenotypes to promote anti-tumoral functions. Translating findings from preclinical models to human patients is further confounded by inter-patient heterogeneity. This variability not only complicates the development of a universally applicable diagnostic paradigm but also impedes the creation of standardized targeted therapeutic agents. Single-cell datasets often generate vast hypotheses, but functional validation in physiologically relevant systems is time-intensive and technically demanding. Notably, murine studies may not fully recapitulate human clinical trials. In addition to these, the intricate technical requirements of scRNA-seq pose a significant barrier to its widespread clinical implementation.

These technological and clinical constraints underscore the imperative for a more intensive investigation of this area. Future investigations should prioritize the establishment of standardized annotation criteria and the integration of multi-omics datasets. Such efforts are essential for providing a more holistic understanding of TAM heterogeneity. As an illustration, the development of a large-scale and in-depth pan-cancer atlas of TAMs has described the complicated spectrum of TAMs. This resource not only serves as a comprehensive benchmark for subsequent research but also catalyzes more detailed mechanistic investigations.^84^

Ultimately, bridging the gap between fundamental research and clinical practice necessitates concerted endeavors to translate scientific insights into actionable strategies that can improve patient outcomes. This demand approaches such as leveraging inducers to reprogram pro-tumoral TAM subtypes into their anti-tumoral counterparts. Simultaneously, strategies aimed at expanding the population of tumoricidal TAM subtypes while impairing the pro-tumoral ones hold significant promise. Advancements in drug delivery technologies, including nanoparticle-based systems and chimeric antigen receptor (CAR)-macrophages, have spurred the emergence of novel TAM-targeted therapeutic modalities. Notably, a cutting-edge CAR-M approach targeting human epidermal growth factor receptor 2 (HER2) and CD47 has been successfully developed for the treatment of OC.^85^ This exemplifies the potential of engineered macrophages to precisely modulate TAM functions and enhance anti-tumor immunity, representing a significant stride toward personalized cancer therapy.

In conclusion, exploring novel TAM subtypes is essential for precision tumor oncology and will offer more realizable strategies for gynecological cancers.

## Acknowledgments

This work was funded by a grant from the 10.13039/501100001809National Natural Science Foundation of China (Grant No. 82273007 to Shu Zhang).

## Author contributions

Conceptualization, Shirong Zou; writing – original draft: Shirong Zou; visualization, Shirong Zou; writing – review and editing, Shu Zhang; supervision, Shu Zhang.

## Declaration of interests

The authors declare no competing interests.
